# Comparison of early hemodynamic performance of 19 mm aortic valve bioprostheses in patients with small aortic annulus

**DOI:** 10.1186/1749-8090-10-S1-A193

**Published:** 2015-12-16

**Authors:** Satoru Domoto, Kazuhiko Uwabe, Hiroyuki Koike, Mimiko Tabata, Hiroyuki Nakajima, Hiroshi Niinami

**Affiliations:** 1 Department of Cardiovascular Surgery, Saitama International Medical Center, Saitama Medical University, Saitama, 350-1298, Japan

## Background/Introduction

A possible problem in aortic valve replacement (AVR) for patients with a small aortic annulus is prosthesis-patient mismatch (PPM). Although larger size prostheses have been well studied, the hemodynamics of 19 mm bioprostheses have been reported in only a small number of patients.

## Aims/Objectives

This study aims to compare the early hemodynamic performance of the new Trifecta valve with others in the 19 mm smallest size.

## Method

We retrospectively evaluated100 patients who underwent AVR with 19 mm bioprosthesis (Trifecta valve in 33, Magna Ease valve in 47, Mosaic Ultra valve in 20) at Saitama International Medical Center between April 2012 and August 2014. Hemodynamic performance was evaluated by transthoracic echocardiography at discharge and 1-year follow-up.

## Results

Preoperative characteristics and early clinical outcomes were similar among the 3 groups. Average age was 75.7 years old and average body surface area was 1.40 m2 in all patients. For the Trifecta, Magna, and Mosaic groups, the mean pressure gradient was 10.8 ± 4.4 mmHg, 16.3 ± 5.1 mmHg and 19.2 ± 6.9 mmHg, respectively; the peak pressure gradient was 19.9 ± 7.6 mmHg, 29.2 ± 8.2 mmHg and 35.9 ± 10.9 mmHg, respectively; the EOA was 1.62 ± 0.35 cm2, 1.17 ± 0.22 cm2 and 1.15 ± 0.23 cm2, respectively; the EOAI index was 1.16 ± 0.23 cm2/m2, 0.83 ± 0.19 cm2/m2 and 0.87 ± 0.23 cm2/m2, respectively, at discharge. The MPG and PPG were smallest (p < 0.001) and EOA and EOAI were largest (p < 0.001) with the Trifecta valves among the 3 groups. PPM was not found in patients with the Trifecta valve (p < 0.001). MPG and PPG at 1-year follow-up were a little higher than those at discharge, and the EOA and EOAI were a little smaller than those at discharge.

## Discussion/Conclusion

The new 19 mm Trifecta valve had favorable early hemodynamic performance compared with the conventional valves, and it may be useful for preventing PPM in patients with a small aortic annulus.

